# AKT3 Is a Novel Regulator of Cancer-Associated Fibroblasts in Head and Neck Squamous Cell Carcinoma

**DOI:** 10.3390/cancers13061233

**Published:** 2021-03-11

**Authors:** Hideyuki Takahashi, Susumu Rokudai, Reika Kawabata-Iwakawa, Koichi Sakakura, Tetsunari Oyama, Masahiko Nishiyama, Kazuaki Chikamatsu

**Affiliations:** 1 Department of Otolaryngology—Head and Neck Surgery, Gunma University Graduate School of Medicine, 3-39-22, Showa-machi, Maebashi, Gunma 371-8511, Japan; htakahas@gunma-u.ac.jp (H.T.); koichisakakura@yahoo.co.jp (K.S.); 2 Department of Molecular Pharmacology and Oncology, Gunma University, 3-39-22, Showa-machi, Maebashi, Gunma 371-8511, Japan; srokudai@gunma-u.ac.jp; 3 Division of Integrate Oncology Research, Gunma University Initiative for Advanced Research, 3-39-22, Showa-machi, Maebashi, Gunma 371-8511, Japan; r.kawabata@gunma-u.ac.jp (R.K.-I.); m.nishiyama@gunma-u.ac.jp (M.N.); 4 Department of Diagnostic Pathology, Gunma University Graduate School of Medicine, 3-39-22, Showa-machi, Maebashi, Gunma 371-8511, Japan; oyama@gunma-u.ac.jp

**Keywords:** cancer-associated fibroblast, AKT, head and neck squamous cell carcinoma, immunosuppression, tumor microenvironment

## Abstract

**Simple Summary:**

Cancer-associated fibroblasts (CAFs) promote epithelial-to-mesenchymal transition, angiogenesis, and immunosuppression, resulting in tumor progression. The PI3K-AKT pathway is known to play vital roles in various cellular activities, including proliferation, growth, metabolism, and survival. In the present study, we sought to identify the key regulator of CAFs in head and neck squamous cell carcinoma (HNSCC) and elucidated the vital roles of *AKT3*, one of the AKT isoforms, in CAFs. A loss-of-function approach revealed that *AKT3* in CAFs promoted their myofibroblastic phenotype and immunosuppressive characteristics. Moreover, the infiltration of AKT3-positive CAFs into tumors was positively correlated with that of various immune cells and an unfavorable prognosis in HNSCC patients. Our findings suggest that AKT3 is a potential biomarker to evaluate the CAF activity and immunosuppressive microenvironment in HNSCC. Furthermore, AKT3 is a potential target for cancer therapy that inhibits the pro-tumoral function of CAFs.

**Abstract:**

Cancer-associated fibroblasts (CAFs) play vital roles in tumor progression by promoting epithelial-to-mesenchymal transition, angiogenesis, and immunosuppression. In the present study, we sought to identify the key regulators of the pro-tumoral functions of CAFs in head and neck squamous cell carcinoma (HNSCC). mRNA expression data obtained from The Cancer Genome Atlas revealed that CAF-specific mRNA expression correlated with genes that relate to an immunosuppressive microenvironment in a HNSCC cohort. RNA sequencing of CAFs and normal fibroblasts isolated from HNSCC specimens identified 1127 differentially expressed genes (DEGs) and several upregulated pathways in CAFs. Among the 1127 DEGs, we identified 13 immune function-related genes and focused on *AKT3* as a potential regulator of CAFs. The targeted depletion of *AKT3* in CAFs revealed that *AKT3* promotes their myofibroblastic phenotype. *AKT3*-transduced CAFs exhibited downregulated the expression of immunosuppressive cytokine genes, impairing T-cell suppression and pro-tumoral macrophage induction. The immunohistochemistry of 72 HNSCC patients showed that AKT3 expression in CAFs positively correlated with tumor infiltration by CAFs, tumor-associated macrophages, dendritic cells, and T cells. Moreover, AKT3 expression in CAFs was an independent prognostic factor for overall survival. In conclusion, AKT3 is a potential target for cancer therapy that inhibits the pro-tumoral function of CAFs and reverses CAF-mediated immunosuppression.

## 1. Introduction

Approximately 650,000 new cases of head and neck squamous cell carcinoma (HNSCC) are diagnosed annually [1]. Despite continuous improvements in treatment regimens, the five-year overall survival rate remains approximately 50% [2], and new therapeutic avenues for HNSCC continue to be explored. During the last decade, some breakthrough HNSCC treatment modalities, including immunotherapies and targeted molecular therapies, have been achieved. In particular, emerging immunotherapy regimens targeting immune checkpoint programmed death 1 (PD-1) have created a new paradigm for the treatment of patients with HNSCC, especially patients with recurrent/metastatic disease [3,4]. However, these advances have only translated into a survival benefit for 20–30% of HNSCC patients, suggesting that a better understanding of the interactions between host immunity and HNSCC tumor microenvironment (TME) is needed. In the TME of HNSCC, tumor cells evade immunosurveillance by several mechanisms, including the downregulation of human leukocyte antigen expression and suppression of tumor-infiltrating lymphocyte functions [5]. In addition to tumor cells, stromal cells also play an important role in tumor cell evasion of the host immune system. Based on accumulating evidence, a combined positive score (CPS), the scoring system for programmed death-ligand 1 (PD-L1) expression that incorporates expression by tumor cells and infiltrating immune cells, is recommended for HNSCC patients who will receive the PD-1 inhibitor pembrolizumab to select treatment modalities [4].

Various types of stromal cells contribute to tumor progression within the TME. Cancer-associated fibroblasts (CAFs) have been recognized as one key component of the TME because of their pivotal roles in promoting epithelial-to-mesenchymal transition, angiogenesis, and the evasion of host immune responses [6,7,8]. We previously demonstrated the CAF immunosuppressive functions in HNSCC [9,10]. CAFs isolated from HNSCC surgical specimens suppress T-cell immunity by suppressing T-cell proliferation, inducing T-cell apoptosis and activating regulatory T cells in vitro. These CAFs also induce M2-like macrophages that strongly suppress T-cell proliferation in vitro. In patients with HNSCC, CAF infiltration into the microenvironment correlates with M2-like macrophage infiltration, worsening the prognosis. CAF immunosuppressive functions have been reported in various cancers, including lung squamous cell carcinoma, melanoma, and pancreatic cancer [11,12,13]. Therapeutic strategies targeting CAFs may improve immunotherapy outcomes by providing a more efficient regulation of a tumor’s immunosuppressive microenvironment.

The phosphoinositide 3-kinase (PI3K)-AKT pathway plays a vital role in various cellular activities, including proliferation, growth, metabolism, and survival, in many cancer types [14,15,16]. In HNSCC, the PI3K-AKT pathway is upregulated in over 90% of cases through mechanisms including epidermal growth factor receptor activation, PI3K overexpression, phosphatase and tensin homolog mutations, and phosphatidylinositol-4,5-bisphosphate 3-kinase catalytic subunit alpha (PIK3CA) mutations or amplifications [17]. AKT is a serine/threonine protein kinase that modulates various cellular responses via the PI3K-AKT pathway. AKT consists of three isoforms: AKT1, AKT2, and AKT3. The overactivation of AKT has been reported in various cancers; however, the specific role of each AKT isoform in malignant diseases still remains unclear [18,19].

The purpose of the present study was to investigate the relationship between CAFs and the immune microenvironment and to elucidate a CAF-specific gene signature by a transcriptome analysis. We demonstrated a correlation between CAF markers and the immunosuppressive microenvironment by analyzing a public database. We next performed RNA-sequencing and identified genes and pathways upregulated in CAFs. Finally, we demonstrated that pharmacological AKT3 modulation may allow CAF reprogramming of an antitumor phenotype.

## 2. Materials and Methods

### 2.1. The Cancer Genome Atlas (TCGA) Dataset

RNA-sequencing data (Illumina Hiseq RNAseq V2, RSEM normalized) and clinical information from HNSCC patients were downloaded from TCGA Research Network (TCGA Provisional version updated in 2016, http://cancergenome.nih.gov/, accessed on 6 May 2020). These datasets consisted of 98 human papillomavirus (HPV)-positive HNSCC samples, 424 HPV-negative HNSCC samples, and 44 normal tissue samples. mRNA expression levels were log_10_-transformed. Receiver operating characteristic curves were plotted to determine the optimal cut-off point to perform a survival analysis for fibroblast activation protein (FAP) expression.

### 2.2. Isolation of Fibroblasts

CAFs and normal fibroblasts (NFs) were isolated from surgical specimens from five newly diagnosed HNSCC patients who did not receive chemotherapy, immunotherapy, or radiotherapy, according to a method we previously described [9]. Briefly, tumor tissues and their normal counterparts obtained from surgical tissues were processed in penicillin, streptomycin, and fungizone. Tissues were sliced into tiny pieces and plated in 6-well tissue culture plates. After culturing cells through several passages, fibroblasts were isolated and used for subsequent assays. Fibroblast identity was confirmed by the detection of fibroblast activation protein (FAP), CD90, and α-smooth muscle actin (α-SMA) using flow cytometry [9]. This study was approved by the Institutional Review Board of Gunma University (No. 12–12) and was performed in line with the Declaration of Helsinki of 1996. All patients provided written informed consent.

### 2.3. RNA Sequencing (RNA-seq)

Total RNA was isolated from fibroblasts using a RNeasy Mini Kit (QIAGEN, Hilden, Germany). RNA quality was evaluated using an Agilent Bioanalyzer (Agilent Technologies, Santa Clara, CA, USA) that confirmed that all RNA samples were of high quality (RNA integrity numbers > 7.0). A TruSeq Stranded mRNA Sample Prep Kit (Illumina, San Diego, CA, USA) was used to generate sequencing libraries of barcoded fragments. Libraries were subjected to paired-end sequencing, yielding 47 base pair (bp) reads on the NextSeq500 System (Illumina) using a NextSeq500 High Output v2 Kit (Illumina). Reads were aligned to the University of California, Santa Cruz, reference human genome 19 (hg19) using Spliced Transcripts Alignment to a Reference (STAR) software v2.5.2b (DNASTAR, Madison, WI, USA). Differentially expressed genes (DEGs) were detected using the TCC-DEGES edgeR-edgeR pipeline [20]. A gene set enrichment analysis (GSEA, using GSEA v4 from the Broad Institute, Cambridge, WA, USA) was used to investigate the pathways upregulated in CAFs. The hallmark pathways and c2 canonical pathways were examined. For each gene set, the normalized enrichment score (NES), *p*-value, and false discovery rate (FDR) *q*-value were calculated to test the enrichment significance. In addition, 1127 DEGs were filtered for entries on a list of 770 genes that relate to adaptive and innate immune responses. This gene list was based on the nCounter^®^ PanCancer Immune Profiling Panel (NanoString, Seattle, WA, USA).

### 2.4. Transfection

shRNAs for luciferase (LUC), AKT3, and PIK3CA were transfected into isolated CAFs from surgical tongue cancer specimens. The shRNA oligonucleotides were annealed at 90 °C for 15 min, 70 °C for 30 min, and 25 °C for 30 min, then cloned into the pLKO.1 puro lentiviral shRNA expression vector between the AgeI and EcoRI sites. Oligonucleotide target sequences used for the shRNAs were as follows: AKT3#4, 5′- GTAGTCCAACTTCACAAAT -3′ and AKT3#5, 5′- GATGTGGATTTACCTTATC-3′. Culture medium was collected from semiconfluent cultures 48 h after medium was changed and stored at −80 °C.

### 2.5. RT-PCR

Total RNA was extracted from cultured cells using a RNeasy mini kit (QIAGEN). RT-PCR was performed in triplicate using a Power SYBR Green RNA-to-CT 1-Step Kit and an Applied Biosystems StepOne instrument (Thermo Fisher Scientific, Waltham, MA, USA). Product specificity was assessed using melting curves recorded at the end of every run. Glyceraldehyde-3-phosphate dehydrogenase (GAPDH) was used as an internal control. Relative expression levels were calculated by the 2-ΔΔCt method, in which Ct represents the threshold cycle. The PCR primers used in this study are shown in Table S4.

### 2.6. Preparation of Peripheral Blood Mononuclear Cells and Isolation of CD14+ Cells

Peripheral blood mononuclear cells (PBMCs) were obtained from healthy donor blood by density gradient centrifugation in a Ficoll-Paque PLUS (GE Healthcare, Chicago, IL, USA). CD14+ cells were then isolated using the EasySep™ Human CD14-Positive Selection Kit II (STEMCELL Technologies, Vancouver, BC, Canada) according to the manufacturer’s protocols.

### 2.7. Cell Proliferation Assay

Transfected CAFs were harvested, and 1 × 10^4^ CAFs/well were plated and incubated in 96-well plates. After incubation, 20 µL of CellTiter 96^®^ AQueous One Solution Reagent (Promega, Madison, WI, USA) was added to each well and incubated for 2 h. Absorbance at 490 nm was then measured using an iMark™ Microplate Absorbance Reader (Bio-Rad, Hercules, CA, USA).

### 2.8. Migration Assay

Migration of transfected CAFs was measured using Transwell inserts (Corning, NY, USA). A total of 2 × 10^4^ transfected CAFs were seeded in the upper chamber with serum-free media. The lower chamber contained Dulbecco’s Modified Eagle Medium (DMEM) supplemented with 10% FCS, 100 units/mL of penicillin, and 100 μg/mL of streptomycin. After 48 h of incubation, nonmigrated cells were removed using a cotton swab, and cells that migrated were stained with 0.1% crystal violet. CAFs that migrated were counted in five different fields under a microscope (magnification 100×). The effect of transfected CAFs on monocyte migration was evaluated using the same system. A total of 3 × 10^4^ CD14+ cells from healthy donors were seeded in the upper chamber in serum-free media. The lower chamber was filled with a transfected CAF-conditioned medium (diluted by half with RPMI supplemented with 10% FCS, 100 units/mL of penicillin, and 100 μg/mL of streptomycin). After 24 h of incubation, cells that had migrated were counted (magnification 200×).

### 2.9. T-cell Proliferation Assay

Healthy donor PBMCs were labeled with 1-µM carboxyfluorescein succinimidyl ester (CFSE, Thermo Fisher Scientific) according to the manufacturer’s protocols. CFSE-labeled PBMCs (1 × 10^5^) were plated into 96-well plates in the presence of transfected CAF conditioned medium (diluted by half with RPMI supplemented with 10% FCS, 100 units/mL of penicillin, and 100 μg/mL of streptomycin) and an anti-CD3/anti-CD28 stimulus (Dynabeads™ Human T-Activator CD3/CD28 for T-cell expansion and activation; Thermo Fisher Scientific), followed by incubation for 7 days. Cells were then harvested and stained with allophycocyanin (APC)-anti-CD3 mAb (BD Bioscience, San Jose, CA, USA). T-cell proliferation was analyzed by the dilution of CFSE staining intensity using flow cytometry. Proliferated cells were gated based on nonstimulated control cells.

### 2.10. Macrophage Induction Assay

CD14+ cells isolated from healthy donors were cultured in transfected CAF conditioned medium (diluted by half with RPMI supplemented with 10% FCS, 100 units/mL of penicillin, and 100 μg/mL of streptomycin) for 7 days. Medium was replaced with fresh medium on day 4.

### 2.11. Flow Cytometry Staining and Analysis

A single cell suspension was incubated with BD Fc Block (BD Bioscience) and fluorescently labeled antibodies at 4 °C for 1 h. Antibodies to cell surface markers, including CD206 (MMR), CD163 (GHI/61), CD80 (2D10), CD86 (IT2.2), HLA-DR (LN3), CD3 (UCHT-1), CD4 (RPA-T4), and CD8 (RPA-T8), were purchased from BioLegend (San Diego, CA, USA), Thermo Fisher Scientific, and BD Biosciences. For intracellular staining, cells were fixed and permeabilized using Foxp3/Transcription Factor Staining Buffer Set (Thermo Fisher Scientific), then incubated with a fluorescently labeled antibody against CD68 (Y1/82A, BioLegend). Multicolor flow cytometry was performed using a BD FACSVerse Flow Cytometer. Acquired data were analyzed using FlowJo (TreeStar, Ashland, OR, USA).

### 2.12. Preparation of Patient Tissue Samples for Immunohistochemistry

Immunohistochemistry (IHC) was performed with 72 HNSCC samples resected from primary tongue cancers. All samples were obtained from patients who underwent surgery without preoperative chemotherapy, immunotherapy, or radiotherapy at Gunma University Hospital (Maebashi, Japan) between November 2000 and January 2012. Clinical information was collected from electronic medical records. Pathological tumor node metastasis classification was established using the 7th edition of the International System for Staging, adopted by the American Joint Committee on Cancer and the Union for International Cancer Control.

### 2.13. Immunohistochemistry

Samples were fixed in 10% formaldehyde and processed for paraffin embedding. Serial histological sections (thickness of 5 µm) were deparaffinized in xylene and hydrated in increasingly dilute ethanol. Antigen retrieval was performed by the application of Proteinase K (Dako, Glostrup, Denmark) at room temperature for 5 min for CD68 staining or by autoclaving at 121 °C for 20 min in citrate buffer (pH 6.0) for the others. Endogenous peroxidase was blocked with 3% H_2_O_2_; then, nonspecific binding sites were blocked with 1% BSA/5% normal horse serum at room temperature for 30 min. Slides were incubated at 4 °C overnight with the primary antibodies listed in Table S7, followed by Labeled Polymer-HRP anti-mouse/rabbit (Dako) at room temperature for 45 min. Reaction products were detected using 3.3′-diaminobenzidine (DAB; Dako), followed by counterstaining with Mayer’s hematoxylin (FUJIFILM Wako Pure Chemical Corporation, Osaka, Japan). After dehydration in increasingly concentrated ethanol, slides were mounted with the nonaqueous mounting medium DPX (Merck, Darmstadt, Germany).

### 2.14. Evaluation of Immunohistochemistry

Slides were evaluated by two independent investigators in a blinded manner using an Axioscope (Carl Zeiss Microscopy GmbH, Jena, Germany) light microscope. Images were acquired using AxioVision LE. CAFs were identified by a spindle-shaped structure with an elongated nucleus in combination with positive α-SMA staining. The proportion of AKT3-positive CAFs ≥ 1% was defined as positive. The proportion of α-SMA-positive fibroblasts was classified into four grades: negative (0), no CAFs, scanty (1), a small number of scattered CAFs, focal (2), concentrated CAFs with irregular and noncontinuous foci, and abundant (3), concentrated CAFs with extensive, continuous foci based on a previously published method [21,22]. Each specimen was scored at the highest grade throughout the entire tumor invasive stroma at ×100 magnification. More than four areas of a representative field adjacent to cancer cells were counted at ×200 magnification for CD68+ and CD163+ macrophages. More than five areas of a representative field were counted for CD1a+ dendritic cells and CD56+ natural killer cells at ×200 magnification. The infiltration of CD3+ T cells in more than five ×400 fields (HPF) was considered grade 1 (<10 positive cells⁄HPF), grade 2 (10–30⁄HPF), grade 3 (31–100⁄HPF), and grade 4 (>101⁄HPF). An average of multiple counted areas was calculated. 

### 2.15. Statistical Analysis

Data were analyzed using GraphPad Prism version 6.0 for Windows (GraphPad Software, San Diego, CA, USA) and R (The R Foundation for Statistical Computing, Vienna, Austria). The *t*-test, chi-square test for independence, and Fisher’s exact test were used to examine the differences in the continuous and categorical variables. Two-sided *p*-values < 0.05 were considered statistically significant. Survival curves were analyzed by the Kaplan–Meier method, and compared using the log-rank test. Univariate and multivariate regression analyses were performed using Cox’s proportional hazards model. Variables with *p*-values < 0.05 were included in subsequent multivariate analyses.

## 3. Results

### 3.1. Expression of CAF Marker Mrnas Correlates with Immunosuppressive Microenvironment in Large HNSCC Cohort 

**Figure 1:**
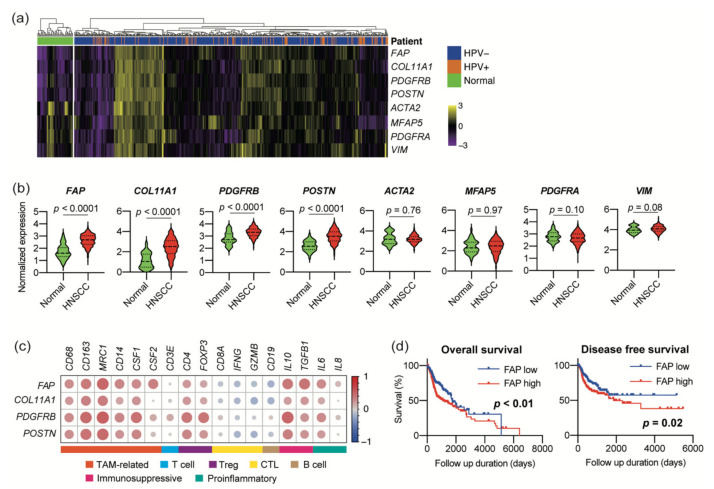
mRNA expression of cancer-associated fibroblast (CAF)-specific markers that correlate with the immunosuppressive tumor microenvironment in head and neck squamous cell carcinoma (HNSCC). (**a**–**d**), mRNA expression data and clinical information was obtained from The Cancer Genome Atlas (TCGA). (**a**) Heat map of CAF-related genes in 522 HNSCC and 44 normal tissues. (**b**) Log_10_-transformed expression of CAF-related genes displayed in (a). (**c**) Correlation matrix displaying R values for the assessment of the correlation between the normalized expression of CAF-specific markers and that of immune markers across 522 HNSCC tissues. (**d**) Kaplan–Meier survival curves of HNSCC patients based on the fibroblast activation protein (FAP) expression. TAM, tumor-associated macrophage, Treg, regulatory T cell, and CTL, cytotoxic T lymphocyte.

To investigate whether CAF functions correlate with the immune microenvironment in HNSCC, we analyzed the RNA-sequencing data from TCGA. The normalized mRNA expression of general CAF markers, including *FAP*, *ACTA2*, *MFAP5*, *COL11A1*, *VIM*, *PDGFRA*, *PDGFRB*, and *POSTN*, were compared between 44 normal samples and 522 HNSCC tissues to identify the CAF markers upregulated in HNSCC. Among these markers, *FAP*, *COL11A1*, *PDGFRB*, and *POSTN* were more highly expressed in HNSCC samples than in normal samples (Figure 1a,b). We next evaluated the correlation between the expression of four CAF-specific markers and immune markers in 522 HNSCC tissues. The expression of the CAF-specific markers correlated positively with that of *CD68*, *CD163*, *MRC1*, *CD4*, *FOXP3*, *CD14*, *IL10*, *TGFB1*, *IL6*, *CXCL8*, *CSF1*, and *CSF2* and correlated negatively with *CD8A*, *IFNG*, and *GZMB* (Figure 1c and Table S1). The FAP expression correlated with a shorter survival of HNSCC patients (Figure 1d). These results indicate that tumor infiltration by CAFs correlates with the immunosuppressive microenvironment, resulting in poor prognosis.

### 3.2. Sequencing of CAF mRNA Revealed a Distinct Signature of Upregulated Genes

**Figure 2:**
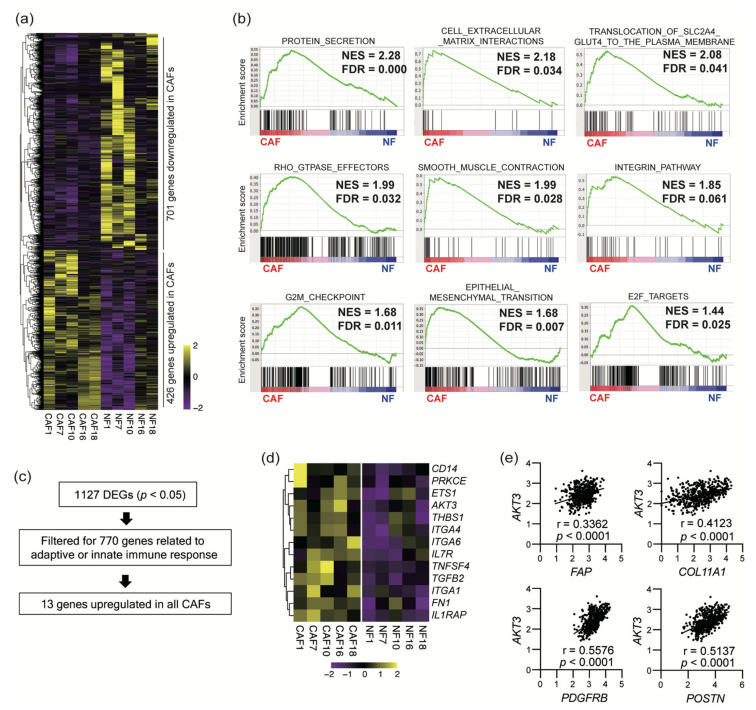
RNA sequencing revealed a CAF-specific gene signature that included immune-related genes. (**a**) Heat map of differentially expressed genes (DEGs) in 5 paired CAF (cancer-associated fibroblast) and NF (normal fibroblast) samples obtained by RNA sequencing (*p* < 0.05). (**b**) Representative gene set enrichment analysis (GSEA) plots of hallmark gene sets and C2 canonical pathways comparing CAF-specific DEGs (FDR < 0.05). (**c**) A total of 1127 DEGs were searched for entries on a list of 770 genes that regulate adaptive or innate immune functions. Thirteen genes were found. (**d**) Heat map of 13 genes that were extracted from the 1127 DEGs in (**d**). (**e**) Correlation between the normalized expression of AKT3 and that of CAF-specific markers that were overexpressed in HNSCC tissues. mRNA expression data was obtained from TCGA.

To characterize the CAF gene expression profiles in detail, we performed RNA sequencing of CAFs and NFs isolated from surgical specimens of five HNSCC patients. CAFs differentially expressed 1127 genes, with 426 genes being upregulated and 701 genes being downregulated (Figure 2a, *p* < 0.05). We further performed a GSEA analysis to elucidate the CAF-specific gene signatures (Figure 2b and Tables S2 and S3). CAFs were characterized by gene signatures involved in protein secretion, cell proliferation, epithelial-to-mesenchymal transition, cell–extracellular matrix interactions, glucose uptake, the RHO-related GTPase regulation of cytoskeletal organization, and smooth muscle construction (FDR < 0.05). These CAF characteristics were consistent with their phenotype as activated myofibroblasts. We also filtered 1127 DEGs for hits from a list of 770 immune-related genes to identify 13 DEGs that regulate the adaptive and innate immune responses to guide further analyses (Figure 1c,d). Among these genes, we focused on *AKT3*. AKT3 expression correlated with that of four CAF-specific genes in 522 HNSCC samples in TCGA cohort (Figure 2e).

### 3.3. AKT3 Depletion Reprogrammed CAF Activity

**Figure 3:**
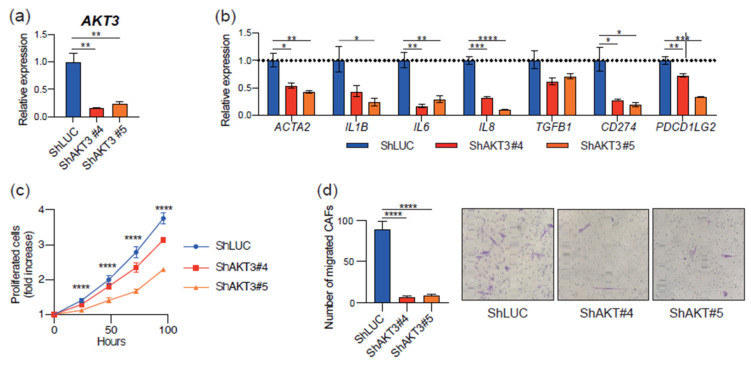
CAF AKT3 is crucial to maintaining their activated phenotype. (**a**) Relative AKT3 mRNA expression in transduced CAFs. (**b**) Relative mRNA expression of immune-related genes in transduced CAFs. (**c**) Proliferation of transduced CAFs. Fold increase compared to 0 h is shown. (**d**) Number of transduced CAFs that migrated through Transwell plates. Representative micrographs of crystal violet-stained migrated cells are shown, 100× magnification. *, *p* < 0.05, **, *p* < 0.01, ***, *p* < 0.001, and ****, *p* < 0.0001.

We next investigated the role of AKT3 in CAF identity and function using a knockdown approach. Two independent shRNAs against *AKT3* (shAKT3#4 and shAKT#5) were transfected into CAFs isolated from a surgical tongue cancer specimen. An shRNA-targeting luciferase (shLUC) was used as a control. As expected, *AKT3* knockdown CAFs expressed less *AKT3* than controls (Figure 3a). *AKT3* knockdown CAFs also exhibited a lower expression of several immune-related genes, including *IL1B, IL6, CXCL8, ACTA2, TGFB1, CD274,* and *PDCD1LG2* (Figure 3b). In addition, *AKT3* knockdown CAFs showed a reduced proliferation (Figure 3c) and migration (Figure 3d) compared with controls. These results suggest that AKT3 expression in CAFs is crucial to maintaining their activated phenotype.

### 3.4. AKT3 Depletion Impaired CAF Immunosuppressive Activity

**Figure 4:**
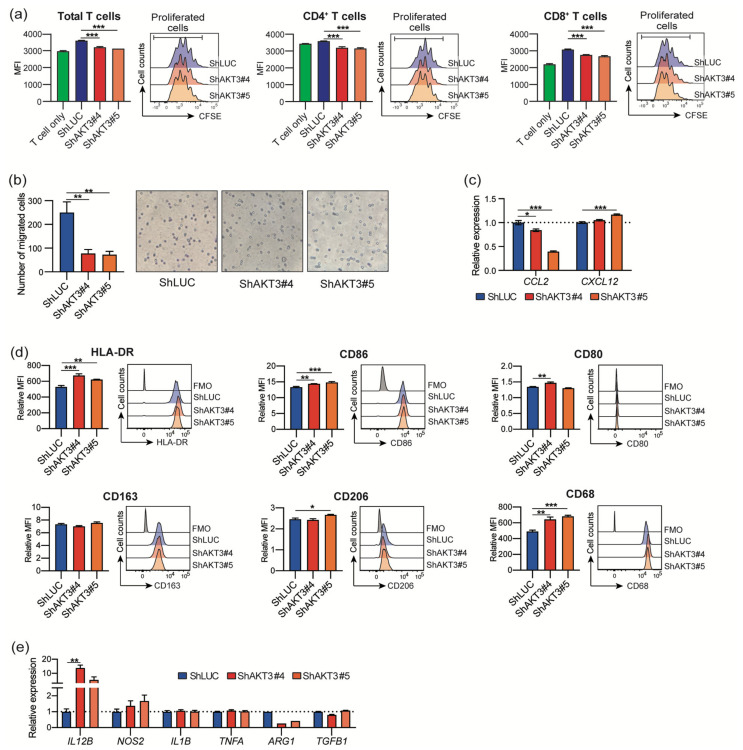
CAF AKT3 is crucial to maintaining their immunosuppressive activities. (**a**) The proliferation of T cells cultured in conditioned medium from transduced CAFs. Lower MFI of CFSE indicates more T-cell proliferation. (**b**) Number of migrated CD14+ monocytes cultured in CAF-conditioned medium. Representative micrographs of crystal violet-stained migrated cells are displayed, magnification 200×. (**c**) Relative mRNA expression of *CCL2* and *CXCL12* in transduced CAFs. (**d**) Expression levels of M1/M2-like markers on macrophages polarized with CAF culture medium in vitro. (**e**) Relative mRNA expression of indicated genes in macrophages polarized with CAF culture medium in vitro. *, *p* < 0.05, **, *p* < 0.01, and ***, *p* < 0.001. MFI, mean fluorescence intensity.

To evaluate the effect of *AKT3* knockdown on the immunosuppressive activity of CAFs, we examined T-cell proliferation, monocyte migration, and macrophage polarization. T cells that were cocultured with conditioned medium from *AKT3* knockdown CAFs proliferated more than control cells (Figure 4a). Fewer CD14+ monocytes migrated into the culture medium from *AKT3* knockdown CAFs than from control cells (Figure 4b). Reduced *CCL2* expression in *AKT3* knockdown CAFs (Figure 4c) was consistent with this result. Macrophages polarized with a conditioned medium from *AKT3* knockdown CAFs expressed more M1-like macrophage markers, including HLA-DR, CD80, and CD86 (Figure 4d), than controls. These macrophages also showed elevated expression of M1-like macrophage genes, including *IL12B* and *NOS2*, and decreased expression of the M2-like macrophage gene *ARG1*. These results indicate that AKT3 plays a vital role in the immunosuppressive characteristics of CAFs. We also knocked down *PIK3CA*, an upstream component of AKT3 signaling. The knockdown of *PIK3CA* in CAFs completely abrogated the CAF viability, and transduced CAFs did not proliferate.

### 3.5. AKT3 Expression in CAFs Correlated with Immune Cell Infiltration into The Tumor Microenvironment

**Figure 5:**
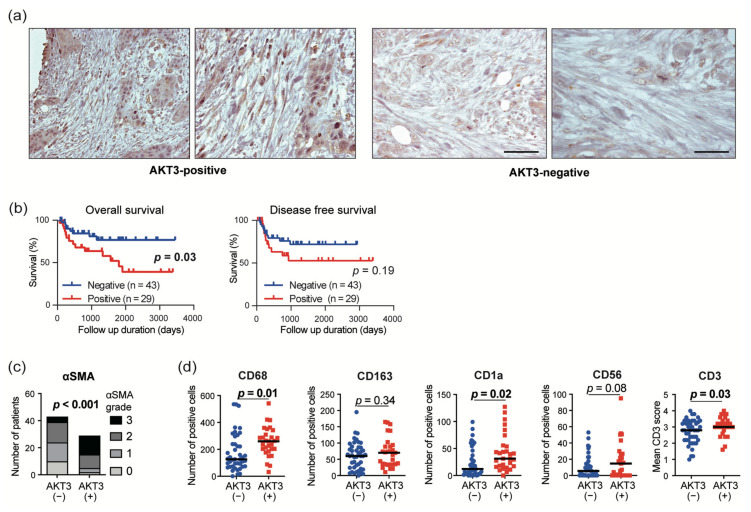
AKT3 expression in CAFs correlates with immune markers and poor prognosis. (**a**) Immunohistochemical staining of head and neck squamous cell carcinoma (HNSCC) surgical specimens with AKT3-positive/negative CAFs; left, 200× magnification, scale bar 100 µm and right, 400× magnification, scale bar 50 µm. CAFs were identified by a spindle-shaped structure with an elongated nucleus in combination with positive α-smooth muscle actin (α-SMA) staining. (**b**) Kaplan–Meier survival curves of patients with HNSCC based on CAF-specific AKT3 positivity. (**c**) Immunohistochemical staining of α-SMA and AKT3 in CAFs. α-SMA grades represent tumor infiltration by CAFs. (**d**) Immunohistochemical staining of immune cell markers and AKT3 in CAFs.

To investigate the clinical significance of AKT3 protein expression in CAFs, we performed IHC for AKT3 in 72 resected surgical specimens from HNSCC patients. The clinical characteristics of the 72 patients are shown in Table S5. Representative AKT3 staining is shown in Figure 5a. We found no relationship between the clinical features, including age, gender, differentiation, lymphatic invasion, vascular invasion, N factor, T factor, and TNM stage, and AKT3 expression in CAFs (Table S6). CAF AKT3 positivity was also compared with α-SMA expression and several immune markers that were also examined by IHC (Figure 5c,d). AKT3 expression in CAFs was significantly positively correlated with the proportion of α-SMA-positive CAFs (*p* < 0.001), number of CD68+ tumor-associated macrophages (*p* = 0.01), number of CD1a+ dendritic cells (*p* = 0.02), and number of CD3+ T cells (*p* = 0.03). We also investigated the PIK3CA expression in CAFs. PIK3CA is one of the catalytic subunits of the phosphoinositide 3-kinases, which are upstream factors in AKT3 signaling. PIK3CA was not expressed in CAFs, but the tumor cells expressed PIK3CA.

### 3.6. AKT3 Expression in CAFs Correlated with a Poor Prognosis

Kaplan–Meier survival analyses were performed to determine the prognostic value of AKT3 expression in CAFs (Figure 5b). The overall survival (OS) was significantly shorter in patients with AKT3-positive CAFs. Disease-free survival (DFS) also trended shorter in patients with AKT3-positive CAFs (not significant). We applied Cox’s proportional hazards regression model to compare the prognostic value of AKT3 expression in CAFs with clinical features (Table 1). Age (*p* = 0.045), differentiation (*p* = 0.038), lymphatic invasion (*p* = 0.006), vascular invasion (*p* = 0.002), TNM stage (*p* = 0.020), and AKT3 expression in CAFs (*p* = 0.037) significantly influenced the OS in the univariate analysis, and both age and AKT3 expression in CAFs were independent prognostic factors in the multivariate analysis. The clinical features, including differentiation (*p* = 0.019), lymphatic invasion (*p* = 0.007), and N factor (*p* = 0.001), significantly influenced DFSin the univariate analysis. No independent prognostic factors were observed for DFS in the multivariate analysis.

## 4. Discussion

Fibroblasts are a major source of connective tissue for the extracellular matrix (ECM) in normal tissue. They are activated to a “myofibroblast” phenotype and play an important role during tissue damage, inflammation, and fibrosis [23]. As cancers are called “wounds that do not heal”, CAFs provide various functions of myofibroblasts, including matrix remodeling, the production of growth factors and cytokines, and metabolic effects [7,8,22]. In the present study, we performed a transcriptome analysis using a public database that suggested a relationship between CAFs and immunosuppression in the tumor microenvironment. CAF RNA sequencing revealed several pathways that are selectively upregulated in CAFs. We also identified 13 CAF-specific genes that influence the immune response. We elucidated the contribution of the expression of one of these genes, *AKT3*, to the myofibroblastic phenotype and immunosuppressive characteristics of CAFs. These findings suggest the viability of a new therapeutic modality that targets CAFs in HNSCC.

We examined the mRNA expressions of eight genes that have been reported as CAF-related markers [24] and identified four markers specific to HNSCC samples in TCGA database. The expression of four CAF-specific markers correlated positively with that of *CD68*, *CD163*, *MRC1*, *IL10*, and *TGFB1*, which are markers that characterize tumor-associated macrophages (TAMs) [25,26]. CAF-specific marker expressions also exhibited a positive correlation with *CSF1* and *CSF2*, which promote TAM infiltration. TAMs have various protumoral functions, including the induction of an immunosuppressive microenvironment, promotion of angiogenesis, and enhancement of tumor invasion. We and others have reported that CAFs induce immunosuppressive M2-like TAMs through the production of cytokines, including IL-6, IL-8, IL-10, TGF-β, GM-CSF, and M-CSF [10,27,28,29,30]. In line with this knowledge, the expression of these cytokines also correlated with that of CAF markers in TCGA. By contrast, the expression of CAF-specific markers negatively correlated with that of *CD8A*, *IFNG*, and *GZMB*. The expression of these markers indicates a tumor infiltration by cytotoxic CD8^+^ T cells and other effector cells. This result is consistent with accumulating evidence that shows a dysfunction of cytotoxic T cells mediated by CAFs [9,31]. Moreover, the expression of CAF-specific markers was positively correlated with that of regulatory T-cell (Treg) markers *CD4* and *FOXP3*. Tregs are recognized as an immunosuppressive subset of CD4+ T cells [32]. Based on the above, TCGA analysis revealed a correlation between tumor infiltration by CAFs and the immunosuppressive microenvironment in a large HNSCC cohort.

We performed RNA sequencing to compare the gene expression profiles of CAFs and NFs and identified various differentially expressed genes and pathways upregulated in CAFs. The most upregulated pathway in CAFs by the GSEA analysis was a hallmark protein secretion. This is in concordance with the accumulating evidence indicating the active secretion of various growth factors and cytokines by CAFs. Since activated RHO GTPases in fibroblasts reportedly promote SCC invasion [33], upregulation of the cell–extracellular matrix interaction pathway and RHO GTPase-mediated pathways may regulate ECM deposition and remodeling, resulting in tumor cell invasion and metastasis. Upregulation of the pathways specific to myofibroblasts, such as smooth muscle contractions, regulation of the actin cytoskeleton, and focal adhesion, supports a myofibroblast-like CAF phenotype [23,34]. Recent single-cell RNA sequencing studies have indicated several distinct CAF subpopulations in pancreatic ductal adenocarcinoma, including myofibroblastic CAFs, inflammatory CAFs, and antigen-presenting CAFs [34,35]. Our bulk RNA sequencing results suggest that CAFs in HNSCC mainly comprise myofibroblastic CAFs that play key roles in HNSCC progression. The upregulation of other pathways such as the G2M checkpoint is also consistent with the proliferative features of myofibroblastic CAFs in HNSCC.

In addition to the pathway analysis, we identified 13 CAF DEGs that regulate adaptive and innate immune responses. We focused on *AKT3* for further analysis, because PI3K-AKT signaling is attracting attention as one of the most hyperactivated signaling pathways in many types of cancers [14,15,16]. AKT regulates various cellular processes, including apoptosis, proliferation, and survival, via the phosphorylation of substrates. There are three mammalian isoforms of AKT: AKT1, AKT2, and AKT3. AKT is considered a candidate for cancer therapy because of its upregulation and vital roles in modulating cancer cell proliferation and survival in many types of cancers [18,19]. Interestingly, several studies have suggested that increased AKT activity in CAFs induces tumor cell invasion in a keratinocyte growth factor (KGF)-dependent manner [36,37]. Since AKT3 was the only isoform elevated in CAFs among the AKT isoforms, we further examined the role of *AKT3* expression in CAFs. We performed *AKT3* knockdown in CAFs to investigate the role of AKT3 in regulating CAF activity. *AKT3* knockdown in CAFs decreased *ACTA2* expression, suggesting that AKT3 controls the CAF myofibroblast-like phenotype. *IL1B* and *TGFB1* expression were also decreased in transduced CAFs. These cytokines play important roles in CAF generation and activation [38]. *AKT1* is reported to be a strong regulator of *ACTA2* in fibroblasts freshly isolated from skin with systemic sclerosis [39]. These results suggest that the activation of the AKT signaling pathway is a key regulator of the myofibroblastic phenotype. The reduced proliferation and migration of *AKT3*-transduced CAFs also supports a myofibroblastic CAF phenotype. *AKT3* knockdown in CAFs downregulated various immunosuppressive genes. We previously reported that increased TGF-β production and the upregulated expression of both PD-L1 and PD-L2 in CAFs contributed to the suppression of T-cell proliferation [9]. The downregulation of these immunosuppressive factors might lead to the increased proliferation of T cells cultured in a conditioned medium of *AKT3* knockdown CAFs compared to control CAF-conditioned medium. In addition, the downregulation of *IL6*, *IL8*, and *TGFB1* in *AKT3* knockdown CAFs might polarize macrophages toward the M1-like phenotype, as these cytokines induce M2-like macrophages. These results indicate that AKT3 plays a vital role in regulating the immunosuppressive activity of CAFs. Since AKT is recognized as playing a vital role in cancer progression, AKT3 inhibition has a therapeutic potential by inhibiting both cancer cells and CAFs. The limitation of the present study is that we were not able to investigate the difference between *AKT3* knockdown and *PIK3CA* knockdown on CAFs to clarify whether AKT3 is the only downstream effector of the phenotype observed in *PIK3CA* knockdown cells or if *AKT3* partially contributes to complex PI3K-AKT signaling. The knockdown approach of *AKT1/AKT2* would provide more details about this point.

In clinical samples from 72 HNSCC patients, AKT3 protein expression in CAFs correlated positively with myofibroblastic CAF infiltration into the tumor microenvironment. This suggests that AKT3 may contribute to determining the myofibroblastic fate of CAFs. Moreover, AKT3 expression in CAFs correlated with an elevated infiltration of CD68+ TAMs but did not correlate with an infiltration of CD163+ TAMS. CD68 is a pan-macrophage marker, while CD163 is prominent on M2c-like macrophages. TAMs are a heterogeneous population comprising various subpopulations. M2c-like TAMs are a subpopulation involved in immunoregulation and tissue remodeling [40]. Our results suggest that CAFs that express AKT3 induce tumor infiltration by other TAM phenotypes. The further characterization of TAM phenotypes induced by AKT3-expressing CAFs is needed. The expression of AKT3 in CAFs also correlated with elevated counts of CD1a+ dendritic cells and CD3+ T cells. Dendritic cells are called professional antigen-presenting cells and play a strong role in presenting tumor antigens to T cells. The expression of AKT3 in CAFs might be elevated as a result of activated tumor antigen presentation to facilitate protumoral TME. Of note, the survival analysis showed that CAF AKT3 expression was an independent prognostic factor for the OS, whereas the other variables were not, except for age. The infiltration of AKT3-positive CAFs could be a new prognostic marker for HNSCC.

## 5. Conclusions

We demonstrated that *AKT3* is a key regulator of the myofibroblastic phenotype and immunosuppressive functions of CAFs. Our findings may inform of the development of a novel therapeutic strategy that targets CAFs by regulating AKT3.

## Figures and Tables

**Table 1:** Univariate and multivariate survival analyses of the OS and DFS in 72 HNSCC patients.

Variables		Overall Survival				Disease-Free Survival			
		Univariate		Multivariate		Univariate		Multivariate	
		5-y	*p*-Value	HR (95% CI)	*p*-Value	5-y	*p*-Value	HR (95% CI)	*p*-Value
		Rate (%)				Rate (%)			
Age (years)									
	<71	70.9	0.045	1	0.035	66.3	0.816		
	≥71	47.1		2.769 (1.077–7.118)		59.6			
Gender									
	Male	61	0.866			60.6	0.582		
	Female	69.9				71.5			
Differentiation									
	Well/moderate	66.5	0.038	1	0.166	69.8	0.019	1	0.191
	Poor	40		2.097 (0.734–5.986)		29.6		1.993 (0.709–5.604)	
Lymphatic invasion									
	Negative	75.8	0.006	1	0.099	77.4	0.007	1	0.463
	Positive	40.3		3.514 (0.790–15.630)		40		1.634 (0.440–6.062)	
Vascular invasion									
	Negative	72.9	0.002	1	0.484	71.3	0.062	1	0.583
	Positive	35.9		1.466 (0.502–4.282)		45.1		1.323 (0.487–3.598)	
T factor									
	T1-2	66.6	0.195			66.3	0.657		
	T3-4	24.3				N/A			
N factor									
	N0	67.1	0.062	1	0.932	74.3	0.001	1	0.063
	N1-3	55.8		1.059 (0.282–3.987)		42.6		3.771 (0.930–15.300)	
TNM stage									
	I-II	71.7	0.02	1	0.659	71	0.096	1	0.301
	III-IV	40.8		0.692 (0.135–3.548)		45.7		0.514 (0.146–1.814)	
AKT3 in CAFs									
	Negative	76.7	0.037	1	0.035	72	0.158		
	Positive	46.2		2.860 (1.076–7.600)		51.2			

Abbreviations: OS, overall survival, DFS, disease-free survival, HNSCC, head and neck squamous cell carcinoma, HR, hazard ratio, CI, confidence interval, and CAF, cancer-associated fibroblast.

## Data Availability

The results published here are based in part on the data generated by TCGA Research Network: https://www.cancer.gov/tcga (accessed on 6 May 2020).

## Supplementary Materials

The followings are available online https://www.mdpi.com/2072-6694/13/6/1233/s1: Table S1: Relationship between the expression of CAF-specific markers and immune markers in 522 HNSCC patients from TCGA database, Table S2: Gene Set Enrichment Analysis of the hallmark gene sets and C2 canonical pathways upregulated in CAFs (FDR<0.10), Table S3: Gene Set Enrichment Analysis of the hallmark gene sets and C2 canonical pathways downregulated in CAFs (FDR < 0.10), Table S4: Primers used for qRT-PCR, Table S5: Characteristics of 72 HNSCC patients, Table S6: Relationships between AKT3 expression and clinical parameters in 72 HNSCC patients, Table S7: Antibodies used for immunohistochemistry.
