# Supplementary material for: AKT3 Is a Novel Regulator of Cancer-Associated Fibroblasts in Head and Neck Squamous Cell Carcinoma
Source: Cancers (Basel). 2021 Mar 11;13(6):1233. doi: 10.3390/cancers13061233 (PMC7998536; doi:10.3390/cancers13061233)
Supplement: Supplementary file 1 [file cancers-13-01233-s001.pdf]

cancers

MDPI

Supplementary: AKT3 Is A Novel Regulator of Cancer-Associated Fibroblasts in Head and Neck Squamous Cell Carcinoma

Hideyuki Takahashi, Susumu Rokudai, Reika Kawabata-Iwakawa, Koichi Sakakura, Tetsunari Oyama, Masahiko Nishiyama and Kazuaki Chikamatsu

Table S1. Relationship between expression of CAF-specific markers and immune markers in 522 HNSCC patients from TCGA database.

Genes	*FAP*		*COL11A1*		*PDGFRB*		*POSTN*	
	r-value	*p*-value	*r*-value	*p*-value	*r*-value	*p*-value	*r*-value	*p*-value
CD68	0.292	<0.001	0.233	<0.001	0.302	<0.001	0.288	<0.001
CD163	0.383	<0.001	0.342	<0.001	0.481	<0.001	0.432	<0.001
MRC1	0.500	<0.001	0.371	<0.001	0.542	<0.001	0.482	<0.001
CD3E	−0.016	0.712	−0.040	0.362	0.168	<0.001	0.021	0.633
CD4	0.237	<0.001	0.235	<0.001	0.406	<0.001	0.323	<0.001
FOXP3	0.181	<0.001	0.172	<0.001	0.417	<0.001	0.247	<0.001
CD8A	−0.090	0.039	−0.074	0.090	0.073	0.097	−0.045	0.300
CD19	−0.168	<0.001	−0.168	<0.001	0.051	0.242	−0.083	0.059
CD14	0.329	<0.001	0.189	<0.001	0.325	<0.001	0.262	<0.001
IFNG	−0.132	0.003	−0.142	0.001	−0.072	0.099	−0.155	<0.001
GZMB	−0.117	0.007	−0.187	<0.001	−0.045	0.302	−0.162	<0.001
IL10	0.401	<0.001	0.210	<0.001	0.504	<0.001	0.358	<0.001
TGFB1	0.429	<0.001	0.234	<0.001	0.259	<0.001	0.252	<0.001
IL6	0.304	<0.001	0.165	<0.001	0.306	<0.001	0.223	<0.001
CXCL8	0.108	0.013	0.007	0.873	0.077	0.080	0.014	0.741
CSF1	0.309	<0.001	0.263	<0.001	0.438	<0.001	0.326	<0.001
CSF2	0.354	<0.001	0.010	0.816	0.159	<0.001	0.077	0.081

Abbreviations: CAF, cancer-associated fibroblast; HNSCC, head and neck squamous cell carcinoma; TCGA, The Cancer Genome Atlas.

Table S2. Gene Set Enrichment Anaylisis of hallmark gene sets and C2 canonical pathways up-regulated in CAFs (FDR <0.10).

Gene Set	NES	FDR
HALLMARK_PROTEIN_SECRETION	2.28	0
REACTOME_CELL_EXTRACELLULAR_MATRIX_INTERACTIONS	2.18	0.034
REACTOME_EPHB_MEDIATED_FORWARD_SIGNALING	2.09	0.055
REACTOME_TRANSLOCATION_OF_SLC2A4_GLUT4_TO_THE_PLASMA_MEMBRANE	2.08	0.041
REACTOME_RHO_GTPASES_ACTIVATE_FORMINS	2.04	0.048
REACTOME_RESOLUTION_OF_SISTER_CHROMATID_COHESION	2.04	0.041
REACTOME_MITOTIC_SPINDLE_CHECKPOINT	2.01	0.045
REACTOME_RHO_GTPASES_ACTIVATE_PAKS	2.01	0.039
REACTOME_ANTIGEN_PRESENTATION:_FOLDING_ASSEMBLY_AND_PEPTIDE_LOADING_OF_CLASS_I_MHC	2	0.035
PID_INTEGRIN_A4B1_PATHWAY	2	0.034
REACTOME_RHO_GTPASE_EFFECTORS	1.99	0.032
REACTOME_RHO_GTPASES_ACTIVATE_ROCKS	1.99	0.03
REACTOME_SMOOTH_MUSCLE_CONTRACTION	1.99	0.028
REACTOME_MITOTIC_METAPHASE_AND_ANAPHASE	1.92	0.053
REACTOME_VXPX_CARGO_TARGETING_TO_CILIUM	1.92	0.051
REACTOME_GOLGI_TO_ER_RETROGRADE_TRANSPORT	1.9	0.054
KEGG_FOCAL_ADHESION	1.89	0.058

PID_RHOA_PATHWAY	1.88	0.06
PID_CDC42_PATHWAY	1.88	0.059
KEGG_PATHOGENIC_ESCHERICHIA_COLI_INFECTION	1.87	0.06
SIG_REGULATION_OF_THE_ACTIN_CYTOSKELETON_BY_RHO_GTPASES	1.87	0.057
REACTOME_SYNDECAN_INTERACTIONS	1.86	0.059
BIOCARTA_INTEGRIN_PATHWAY	1.85	0.061
REACTOME_SYNTHESIS_OF_PIPS_AT_THE_EARLY_ENDOSOME_MEMBRANE	1.85	0.058
KEGG_SMALL_CELL_LUNG_CANCER	1.85	0.06
REACTOME_EPH_EPHRIN_SIGNALING	1.81	0.078
REACTOME_RHO_GTPASES_ACTIVATE_CIT	1.8	0.086
REACTOME_NON_INTEGRIN_MEMBRANE_ECM_INTERACTIONS	1.79	0.085
KEGG_REGULATION_OF_ACTIN_CYTOSKELETON	1.79	0.084
REACTOME_ATTENUATION_PHASE	1.79	0.082
REACTOME_HSP90_CHAPERONE_CYCLE_FOR_STEROID_HORMONE_RECEPTORS_SHR	1.78	0.084
REACTOME_COPI_DEPENDENT_GOLGI_TO_ER_RETROGRADE_TRAFFIC	1.78	0.081
HALLMARK_G2M_CHECKPOINT	1.68	0.011
HALLMARK_EPITHELIAL_MESENCHYMAL_TRANSITION	1.68	0.007
HALLMARK_MITOTIC_SPINDLE	1.51	0.028
HALLMARK_UV_RESPONSE_DN	1.5	0.023
HALLMARK_APICAL_JUNCTION	1.49	0.02
HALLMARK_E2F_TARGETS	1.44	0.025
HALLMARK_MTORC1_SIGNALING	1.42	0.027
HALLMARK_ANDROGEN_RESPONSE	1.38	0.031
HALLMARK_MYC_TARGETS_V1	1.26	0.081

Abbreviations: CAFs, cancer-associated fibroblasts; NES, normalized enrichment score; FDR, false discovery rate.

Table S3. Gene Set Enrichment Anaylisis of hallmark gene sets and C2 canonical pathways down-regulated in CAFs (FDR <0.10).

Gene Set	NES	FDR
KEGG_RIBOSOME	−2.54	0
REACTOME_NONSENSE_MEDIATED_DECAY_NMD_INDEPENDENT_OF_THE_EXON_JUNCTION_COMPLEX_EJC	−2.42	0
REACTOME_SELENOAMINO_ACID_METABOLISM	−2.32	0
REACTOME_NONSENSE_MEDIATED_DECAY_NMD	−2.29	0
REACTOME_EUKARYOTIC_TRANSLATION_INITIATION	−2.24	0
REACTOME_SRP_DEPENDENT_COTRANSLATIONAL_PROTEIN_TARGETING_TO_MEMBRANE	−2.15	0
REACTOME_BETA_DEFENSINS	−2.05	0.001
KEGG_METABOLISM_OF_XENOBIOTICS_BY_CYTOCHROME_P450	−2	0.002
REACTOME_COMPLEMENT_CASCADE	−1.96	0.003
REACTOME_REGULATION_OF_EXPRESSION_OF_SLITS_AND_ROBOS	−1.94	0.004
REACTOME_INFLUENZA_INFECTION	−1.93	0.005
BIOCARTA_COMP_PATHWAY	−1.92	0.005
REACTOME_ACTIVATION_OF_THE_MRNA_UPON_BINDING_OF_THE_CAP_BINDING_COMPLEX_AND_EIFS_AND_SUBSEQUENT_BINDING_TO_43S	−1.91	0.005
REACTOME_SYNTHESIS_OF_BILE_ACIDS_AND_BILE_SALTS_VIA_27_HYDROXYCHOLESTEROL	−1.87	0.009
REACTOME_RA_BIOSYNTHESIS_PATHWAY	−1.84	0.013
KEGG_DRUG_METABOLISM_CYTOCHROME_P450	−1.84	0.013
KEGG_RETINOL_METABOLISM	−1.83	0.014
BIOCARTA_CLASSIC_PATHWAY	−1.82	0.015
REACTOME_RRNA_PROCESSING_IN_THE_NUCLEUS_AND_CYTOSOL	−1.81	0.017
REACTOME_SYNTHESIS_OF_BILE_ACIDS_AND_BILE_SALTS_VIA_7ALPHA_HYDROXYCHOLESTEROL	−1.8	0.021
REACTOME_FOXO_MEDIATED_TRANSCRIPTION_OF_OXIDATIVE_STRESS_METABOLIC_AND_NEURONAL_GENES	−1.8	0.02
REACTOME_INITIAL_TRIGGERING_OF_COMPLEMENT	−1.8	0.019
REACTOME_DEFENSINS	−1.79	0.02
REACTOME_DIGESTION_AND_ABSORPTION	−1.78	0.022

KEGG_LINOLEIC_ACID_METABOLISM	−1.78	0.022
KEGG_ARACHIDONIC_ACID_METABOLISM	−1.77	0.022
REACTOME_PHASE_I_FUNCTIONALIZATION_OF_COMPOUNDS	−1.76	0.026
REACTOME_RRNA_PROCESSING	−1.75	0.03
KEGG_COMPLEMENT_AND_COAGULATION_CASCADES	−1.74	0.033
KEGG_STEROID_HORMONE_BIOSYNTHESIS	−1.73	0.036
REACTOME_ARACHIDONIC_ACID_METABOLISM	−1.72	0.037
REACTOME_INTERLEUKIN_20_FAMILY_SIGNALING	−1.72	0.037
REACTOME_BIOSYNTHESIS_OF_SPECIALIZED_PRORESOLVING_MEDIATORS_SPMS	−1.72	0.038
REACTOME_PHASE_2_PLATEAU_PHASE	−1.71	0.038
REACTOME_METABOLISM_OF_AMINO_ACIDS_AND_DERIVATIVES	−1.69	0.051
REACTOME_SIGNALING_BY_ROBO_RECEPTORS	−1.67	0.061
REACTOME_TERMINATION_OF_O_GLYCAN_BIOSYNTHESIS	−1.67	0.061
REACTOME_DIGESTION	−1.66	0.065
REACTOME_INTERLEUKIN_10_SIGNALING	−1.66	0.065
REACTOME_ANTIMICROBIAL_PEPTIDES	−1.66	0.066
HALLMARK_INTERFERON_ALPHA_RESPONSE	−1.64	0.028
REACTOME_ACYL_CHAIN_REMODELLING_OF_PC	−1.63	0.087
HALLMARK_BILE_ACID_METABOLISM	−1.61	0.021

Abbreviations: CAFs, cancer-associated fibroblasts; NES, normalized enrichment score; FDR, false discovery rate.

Table S4. Primers used for qRT-PCR.

Gene	Forward Primer	Reverse Primer
ARG1	5′-AAAGGCTGGTCTGCTTGAGAA-3′	5′-GTCATTAGGGATGTCAGCAAAGG-3′
IL10	5′-GAGATGCCTTCAGCAGAGTGAAGA-3′	5′-AGGCTTGGCAACCCAGGTAAC-3′
TGFB1	5′-AGCGACTCGCCAGAGTGGTTA-3′	5′-GCAGTGTGTTATCCCTGCTGTCA-3′
VEGFA	5′-ACTTCCCCAAATCACTGTGG-3′	5′-GTCACTCACTTTGCCCCTGT-3′
TNF	5′-TGCTTGTTCCTCAGCCTCTT-3′	5′-CAGAGGGCTGATTAGAGAGAGGT-3′
IL1B	5′-TCGCCAGTGAAATGATGGCTTA-3′	5′-GTCCATGGCCACAACAACTGA-3′
IL6	5′-AAGCCAGAGCTGTGCAGA TGAGTA-3′	5′-TGTCCTGCAGCCACT GGTTC-3′
CXCL8	5′-GTGCAGAGG GTTGTGGAGAAGTTT-3′	5′-TCACTGG CATCTTCACTGATTCTTG-3′
NOS2	5′-GCCAAGCTGAAATTGAATGAGGA-′3	5′-TTCTGTGCCGGCAGCTTTAAC-3′
IL12B	5′-GGAGCGAATGGGCATCTGT-3′	5′-TGGGTCTATTCCGTTGTGTCTTT-3′
CXCL12	5′-GAGCCAACGTCAAGCATCTCAA-3′	5′-TTAGCTTCGGGTCAATGCACAC-3′
CCL2	5′-CTTCTGTGCCTGCTGCTCATA-3′	5′-CTTTGGGACACTTGCTGCTG-3′
ACTA2	5′-ATTGCCGACCGAATGCAGA-3′	5′-ATGGAGCCACCGATCCAGAC-3′
CD274	5′-CAATGTGACCAGCACACTGAGAA-3′	5′-GGCATAATAAGATGGCTCCCAGAA-3′
PDCD1LG2	5′-TGGAATTGCAGCTTCACCAGATAG-3′	5′-GGCTGTTATTGCTCCAAGGTTCA-3′
GAPDH	5′-GCACCGTCAAGGCTGAGAAC-3′	5′-ATGGTGGTGAAGACGCCAGT-3′

Table S5. Characteristics of 72 HNSCC patients.

	No. (%)
Age, years	
- median	68
- range	33–92
Gender	
- Male	45 (63)
- Female	27 (37)
Differentiation	
- Well/moderate	62 (86)
- Poorly	10 (14)
Lymphatic invasion	33 (46)

Vascular invasion	23 (32)
T factor	
- T1-2	63 (88)
- T3-4	9 (12)
N factor	
- N0	48 (67)
- N1-3	24 (33)
TNM stage	
- I-II	47 (65)
- III-IV	25 (35)

HNSCC, head and neck squamous cell carcinoma.

Table S6. Relationships between AKT3 expression and clinical parameters in 72 HNSCC patients.

Variable	AKT3 in CAFs		
	Negative	Positive	*p*-value
Age (years)			
- <71	24	17	1.00
- ≥71	19	12	
Gender			
- Male	23	22	0.08
- Female	20	7	
Differentiation			
- Well/moderate	36	26	0.71
- Poorly	7	3	
Lymphatic invasion			
- Negative	25	14	0.41
- Positive	18	15	
Vascular invasion			
- Negative	32	17	0.16
- Positive	11	12	
T factor			
- T1-2	40	23	0.14
- T3-4	3	6	
N factor			
- N0	31	17	0.23
- N1-3	12	12	
TNM stage			
- I-II	33	14	0.43
- III-IV	10	15	

Abbreviations: CAF, cancer-associated fibroblast; HNSCC, head and neck squamous cell carcinoma.

Table S7. Antibodies used for immunohistochemistry.

Antibody	Clone	Dilution	Company
AKT3	Polyclonal	1:250	Sigma-Aldrich
PIK3CA	Polyclonal	1:100	Sigma-Aldrich
αSMA	1A4	1:20	R&D Systems
CD68	PG-M1	Ready-to-use	Dako
CD163	10D6	1:200	Leica Biosystems
CD1a	O10	Ready-to-use	Beckman Coulter
CD3	Polyclonal	Ready-to-use	Dako
CD56	123.C3.D5	Ready-to-use	Thermo Fisher Scientific
